# Effects of Heat Stress on the Well-Being, Fertility, and Hatchability of Chickens in the Northern Guinea Savannah Zone of Nigeria: A Review

**DOI:** 10.5402/2011/838606

**Published:** 2011-06-14

**Authors:** J. O. Ayo, J. A. Obidi, P. I. Rekwot

**Affiliations:** ^1^Department of Physiology and Pharmacology, Faculty of Veterinary Medicine, Ahmadu Bello University, Zaria 81001, Nigeria; ^2^National Animal Production Research Institute, Ahmadu Bello University, Zaria 81001, Nigeria

## Abstract

The paper examines heat stress and its adverse effects as a hindrance to profitable poultry production in the tropics, with emphasis on the Northern Guinea Savannah zone of Nigeria. It elucidates the general negative effects of heat stress on physiological parameters of domestic chickens, and the specific impact of the stress on reproduction in the tropics. The deleterious effects are expressed in poor poultry well-being and reproductive performance. It is concluded that measures aimed at alleviating heat stress in domestic chickens must be adopted in order to enhance reproductive and, consequently, efficiency of modern poultry production in the tropics.

## 1. Introduction

The Northern Guinea Savannah zone of Nigeria is located between latitude 11°N, 12′N and longitude 7°E, 8′E, at an elevation of 650 m above the sea level. The zone has an average annual maximum and minimum temperatures of 31.8 ± 3.2°C and 18.0 ± 3.7°C, respectively. The monthly average rainfall during the rainy season (May–October) is 148 ± 68.4 mm (69.2–231.9 mm), while the monthly relative humidity is 71.1 ± 9.7%. The zone is characterized by three seasons: Harmattan (November–February), hot-dry (March–May), and rainy (June–October) seasons [1, 2].

## 2. Effects of Meteorological Elements on Domestic Chickens

The meteorological elements constitute a complex system, which acts upon the body of domestic chickens. Jointly, they are expressed as climate, that is, long-term average conditions, or as weather. They may affect birds outside or inside the poultry houses, and their impact on birds may be beneficial or detrimental, depending on the extent of their variations. Besides, the impact of meteorological elements may be expressed singly or in combinations; for instance, Bianca [3] reported that low ambient temperature plus high air movement (cold) or high ambient temperature and high relative humidity plus solar radiation (heat) exert various effects on animal well-being, demonstrated in neuroendocrine, cardiorespiratory, and behavioural responses. The concern and emphasis for meteorological elements in recent years are due to the fact that they are not constant, but change continuously. Such changes affect the internal environment of birds, including the blood through the nervous and endocrine systems [3–5]. Each season in the zone has its positive or negative effects on livestock production. For example, the Harmattan season has been described as thermally stressful to goats [2], pigs [6], and poultry [7], while the negative impact of environmental stress on poultry is minimum during the rainy season [8–10].

In the tropical environment, meteorological factors exert significant influence on domestic birds. Direct meteorologic factors affecting birds include, especially, high ambient temperature and high relative humidity, resulting in severe heat stress. This is not the case in temperate countries, where animals are predominantly reared intensively and in-door with automatically regulated microclimatic conditions. Consequently, adverse effects of thermal stress are minimum because ambient temperature and relative humidity are auto-regulated constantly. However, in Nigeria, the local poultry species are predominantly subjected to the effects of heat stress because they are reared mainly under the extensive management system, which significantly exposes chickens to the influence of high ambient temperatures and high relative humidity. When protection is provided against heat, it is usually inadequate because it is in form of temporary light shade and radiation shield [9, 11, 12]. The report of Egbunike [13] suggests that the effect of a hot environment on the body of birds depends not only on the nature of the microclimatic factors involved, but also on their intensity. According to Egbunike [13], environment affects adversely the chicken. This observation corroborates the findings of Donald [14] that a greater number of physiological activities undergo specific changes in birds exposed to a hot environment. These facts are further enunciated by the report of Becker et al. [15], who elaborated on the negative impacts of heat stress on the neuroendocrine, cardiovascular, respiratory and behavioural responses adopted by animals to adjust to altered environmental influences. 

In poultry production in the tropical and subtropical regions, heat stress is very common and on the increase. It inflicts heavy economic losses on poultry production as a result of stunted growth [16], decrease in hen-day production [17–21], increased cost of production, high rate of mortality due to depressed immunity, and reproductive failure [10, 21, 22]. In Nigeria, the combination of high ambient temperature and high relative humidity, reaching climax at the onset of the rainy season, constitutes extreme heat stress, which depresses the production of broiler and layer birds. Conversely, the low ambient temperature and low relative humidity, prevailing from November to February during the Harmattan season, especially in the morning and evening hours of the day, enhance egg and broiler production [8, 9]. Heat stress depresses growth rate and production as a result of a down-turn in voluntary feed intake in broilers, implying that thermal loads depress enterocyte proliferation and growth [16]. Sahin et al. [16] also showed that body weight in heat-stressed broilers was significantly lower than in birds administered with antioxidant vitamins A and E. This observation supports the report of Donkoh [23] that antioxidants alleviate heat stress in poultry. Sahin et al. [16] further showed that plasma triiodothyronine and thyroxine, which are important growth promoters in animals, were adversely affected in heat-stressed broiler chickens, thus corroborating earlier findings of Bowen et al. [24], Hillmann et al. [25], and McNabb and King [26] that heat stress is a deterrent to successful and profitable poultry production.

Several research findings on adverse effects of environmental stress on domestic birds showed that plasma level of corticosteroids increases, while that of plasma proteins decreases with marked increase in blood glucose concentration [16, 23, 27, 28]. Thus, it is apparent that the inhibition of growth and production in heat-stressed broiler birds is mediated via the stress hormones, especially the corticosteroids. Normal body functions of birds are efficient if the body temperature is kept constant or at least maintained within narrow limits. If the ambient temperature changes gradually, birds adapt to the changes, but if the changes are rapid and especially accompanied with high relative humidity, they induce heat stress [4]. This observation supports the results obtained by Ayo and Sinkalu [29] and Sinkalu et al. [30] that severe heat stress in pullets occurs during the hot-dry season in the Northern Guinea Savannah zone, as evidenced by a rise in their body temperature values.

 The optimum ambient temperature range for poultry is 12–26°C [31]. At low ambient temperature (7°C and below), birds become depressed, drowsy, and a fall in production level occurs. The negative effects of high ambient temperatures observed during the hot-dry season are even more pronounced than those of the low temperatures. It has been established that at high ambient temperatures, the physiological potentials of birds are sharply reduced [3, 10, 21, 32]. Consequently, if poultry production is to be improved and successfully industrialised in the zone and, indeed, other arid and semiarid regions of the world, further study is required to elucidate the cellular and, especially, the molecular mechanisms of adaptation of poultry breeds to the hot-dry conditions [11, 12].

## 3. Heat Stress and Its Effects on Haematological and Biochemical Parameters

There is a dearth of information on seasonal variations in haematological parameters of domestic chickens in the Northern Guinea Savannah zone of Nigeria. Oladele et al. [5] reported, for the first time, significantly low levels of packed cell volume of 23.73 ± 0.12% in domestic chickens during the hot-dry season, while the values of 24.82 ± 0.49% and 24.63 ± 0.10% were obtained during the Harmattan and rainy seasons, respectively. Haemoglobin values of 8.26 ± 0.16 g % and 8.10 ± 1.12 g% were reported in birds during the Harmattan and rainy seasons, respectively. The low values of haemoglobin and packed cell volume recorded during the hot-dry season in the zone were attributed to heat and nutritional stress, which impair the synthesis of blood cells in birds [28].

Oladele et al. [28] demonstrated that during the hot-dry season meteorological elements were very significantly (*P* < .001) correlated with packed cell volume, haemoglobin, and total protein in the chicken: correlation coefficient (*r*) values obtained between ambient temperature and packed cell volume during the Harmattan, hot-dry, and rainy seasons were 0.874, −0.996, and 0.903, respectively. Furthermore, *r* values of 0.890, −0.996, and 0.985 were obtained when haemoglobin was correlated with ambient temperature values during the Harmattan, hot-dry, and rainy seasons, respectively; indicating that haemoglobin is highly responsive to fluctuations in ambient temperature, with a significant negative response to the deleterious effect of heat stress. Total protein value also had a significant and negative relationship with elevated ambient temperature, and the *r* values obtained during the hot-dry, Harmattan, and rainy seasons were −0.998, 0.943, and 0.999 [28], indicating that heat stress exerts adverse effects on protein synthesis. The result supports the findings of Sahin et al. [16], who demonstrated a significant negative effect of heat stress on total proteins in broiler chickens. Sahin et al. [16] also reported significant negative effects of heat stress (32°C) on serum concentration of some metabolites and minerals in broilers. Serum levels of thyroxine (T_4_) and triiodothyronine (T_3_) were significantly reduced due to a high level of adrenocorticotropic hormone in unsupplemented broiler chickens, when compared to values obtained in birds that received antioxidant feed supplements. Serum calcium and phosphorus levels were observed to be significantly lowered in heat-stressed birds [32], and this paper corroborates the earlier findings of McDaniel et al. [33] who recorded a decrease in serum calcium level in heat-stressed broiler chickens.

## 4. Effects of Heat Stress on Reproduction in Domestic Chickens

### 4.1. Effects of Heat Stress on Egg Quantity and Quality

Ambient temperatures may influence reproductive ability by altering feed intake of chickens. Ayo et al. [21] obtained 20% reduction in feed intake in heat-stressed layer chickens during the hot-dry season, associated with high ambient temperature and high relative humidity which resulted in a significant decrease in hen-day production. The result supports the finding of Abd-Ellah [34], who recorded a significant decrease in hen-day production as a consequence of an appreciable reduction in average daily feed consumption in severe arid conditions of Egypt, when average ambient temperature was 43°C. Khan and Sarda [20] and Simon [35] observed a decrease in voluntary feed intake by birds, attributable to physiological response to heat stress, aimed at reducing the excessive endogenous heat generated in the body due to feed metabolism. The results are in agreement with those of Bollengier-Lee et al. [36], Mahmoud et al. [37], and De-Fariara et al. [19], who demonstrated that heat stress depressed egg production and reduced the external and internal egg qualities. The depression as reported by Mahmoud et al. [37] was due to an imbalance in calcium-estrogen relationship and lowered Haugh unit of the albumen. This implies that high environmental temperature depresses yolk size, albumen consistency, and optimum calcium deposit in the egg shell.

### 4.2. Mechanisms of Action of Heat Stress on Reproduction

Dantzer and Kelly [38] showed that physical and emotional stressors suppress immunity through the activation of cytokine IL-1, which induces fever and reduces feed intake. The finding suggests that cytokine IL-1 also stimulates the hypothalamic-pituitary-adrenal axis and inhibits the hypothalamic-pituitary-gonadal functions, implying that cytokine IL-1 may also mediate some behavioural responses due to heat stress. This observation is, apparently, the closest report on the proximate mechanism of heat-induced infertility in domestic animals, including the domestic chicken. In view of the report of Dantzer and Kelly [38], it is reasonable to conclude that stress due to high environmental temperatures disturbs the pulsatile gonadotrophin-releasing hormone generator frequency, which in turn compromises reproductive functions of the axis, due to heat-induced impairment in the secretion of follicle-stimulating and luteinising hormones in laying birds. Altan et al. [39] demonstrated that heat stress in broilers, induced by high ambient temperature and high relative humidity, results in lipid peroxidation of cytomembranes due to excessive free radical generation. Although the underlying mechanisms of disruption of the brain-reproductive axis may not be fully elucidated in birds, a model of the influence of stress on the disruption of the hormonal regulation of female reproduction in sheep has been reviewed by Dobson et al. [40], which provides some information in this regard.

### 4.3. Losses on Eggs and Laying Birds due to Heat Stress

Reproductive losses due to heat stress are manifested in the percentage of culled birds and mortality due to heat stroke. High environmental temperatures induce heat stress in poultry, manifested in stressful behavioural responses such as panting, elevated respiratory rate, and restlessness, resulting in loss of body fluids (dehydration) [13, 21], which may cause death due to heat stroke. If timely intervention measures are adopted, birds may be salvaged from death, although a high rate of culled birds due to heat stress has been reported. Thus, Ayo et al. [21] recorded 3.7% average mortality in laying birds during the hot-dry season in the Northern Guinea Savannah zone of Nigeria, while Abd-Ellah [34] reported as high as 28% mortality in arid weather conditions of Egypt. The difference in the two values is, apparently, due to the greater severity of heat stress in arid conditions of Egypt, with an average ambient temperature of about 43°C. Furthermore, Ayo et al. [21] obtained a mean cull of 1.7% during the hot-dry season in the Northern Guinea Savannah zone of Nigeria, and the value was lower than that of Abd-Ellah [34], apparently due to the greater severity of heat stress in the arid conditions of Egypt.

### 4.4. Adverse Effects of Heat Stress on Semen Characteristics and Sperm Function

Heat stress affects all phases of semen production in breeder cocks as reported in other species [41]. Although limited high temperature stimulates testicular growth in the early phase and promotes increased semen volume and concentration, a subsequent rise suppresses reproductive capacity as a result of a decrease in seminiferous epithelial cell differentiation, which is manifested in decreased semen quality and quantity with time [10, 33, 42]. Serum calcium and phosphorus levels were observed to be significantly lowered in heat-stressed birds [32, 33]. Transient inward calcium ion currents whose density increased during spermatogenesis, from spermatogonia to early spermatids, have been described [43]. Inhibition of calcium and potassium ion exchange significantly decreased spermatogenesis, implying that distinct expression and noninhibition of ion channels during spermatogenesis may enhance the excitation and differentiation of seminiferous (germinal) epithelium [43, 44], as is characteristic of excitable tissues. It is worthy to note that some of the ion channels regulating ion exchange during the preliminary stages of germinal cell differentiation end up in mature spermatozoa, determining their physiological properties [44]. Therefore, based on the decrease in calcium ion level in the reports of McDaniel et al. [32, 33], it may be concluded that heat stress has deleterious multiple effects on testicular function through inhibition of intracellular ion exchange.

The reproductive performance of the rooster is greatly depressed during environmental stress. Although some results have been obtained on heat stress-induced infertility in exotic and small breeds of chickens, very little research has been conducted on the fertility of modern breeders, exposed to elevated ambient temperatures [10, 32, 33]. In a simulated study on the effects of heat stress on fertility in broiler breeder roosters, McDaniel et al. [33] showed that the broiler breeder contributed more to heat-induced infertility than the female. When the male broiler breeder was exposed to a temperature of 32°C, male fertility declined to 42% and *in vivo* sperm-egg penetration declined to 52%, compared to values obtained from males that were maintained at 21°C. This observation demonstrated a significant inhibition of the rooster's spermatozoa viability through qualitative and quantitative depression in semen characteristics such as spermatozoa motility.

 Heat stress may be responsible for the inhibition of osmotic equilibrium and ionic channels that are key elements in the interplay between spermatozoa, its environment, and the egg, thus disrupting spermatozoa cellular homeostasis, distorting spermatozoa behaviour, and metabolic machinery [45]. In mammals, excessive levels of reactive oxygen species have been significantly correlated with decreased sperm motility [46, 47]. It is known that the influx of intracellular calcium ion is important to spermatozoa flagella motility and to the fusion of acrosome vesicle; any interruption of the critical ionic function(s) frustrates viability and fertility. The report of McDaniel et al. [33] showed that semen characteristics such as consistency, spermatozoa concentration, and seminal volume were depressed by environmental temperatures outside the zone of thermal comfort. 

On the overall, based on the findings of McDaniel et al. [32, 33] and King et al. [48], heat-induced infertility is mediated through any compromise in the fluidity and integrity of spermatozoa cell membranes as well as acrosomal and deoxyribonucleic acid damage as established by Surai [49–51], and the inhibition of expression of hyaluronic acid binding sites as well as acrosomal integrity [52, 53]. The differences found when breeder cocks were exposed to elevated ambient temperatures were not evident, when the female birds alone were exposed to the same high ambient temperatures [33, 34]. Edens [42] reported significant effects of ambient temperature on male fertility, which were evident within 12 hours of challenge at a typical summer temperature of 29°C, although semen characteristics such as semen volume, spermatozoa concentration, and percentage dead spermatozoa were unaffected by the heat treatment. This apparent lack of observable depreciation in semen characteristics obtained in the study of Edens [42] suggests that roosters can adapt to short-term exposure to thermal stress. Thus, physiological changes inimical to testicular functions may not occur in short-term exposure to heat stress. The finding of Edens [42] disagreed with those of McDaniel et al. [32, 33], who subjected roosters to a long-term heat exposure. The depression in *in vivo* sperm-egg penetration and fertility in heat-stressed roosters reported by McDaniel et al. [32, 33] may be due to a decrease in number of spermatozoa stored in the sperm host glands in the hen's reproductive tract [54–56]. In other words, a decrease in oviductal spermatozoa storage results in fewer spermatozoa cells available to bind, penetrate, and fertilize the egg in the infundibulum of the hen as documented by King et al. [48].

In mammals, spermatozoa's binding with uterine epithelial cells is a strong index of spermatozoa viability and fertilizing capacity, implying that spermatozoa attachment to uterine epithelial cells is indicative of normal ultrastructure and mitochondrial membrane potential [57–59]. Töpfer-Petersen et al. [60] and Ardón et al. [61] showed that bound spermatozoa have both prolonged lifespan and better chromatin integrity than unbound spermatozoa cells. It is known that spermatozoa that have been bound temporarily to uterine epithelial cells can pass along the oviduct for fertilization [58]. In this context, it is reasonable to conclude that heat stress in the rooster retards or even prevents important physiological mechanisms, such as sperm-uterine epithelial cells interaction, capacitation, acrosome reaction, and zonal vesicle binding, resulting in depression in fertility. This is, apparently, due to a depletion of endogenous antioxidant milieu in semen, leading to speedy exhaustion of spermatozoa energy reserves. On the other hand, it is likely that exposed spermatozoa were properly stored in the hen's oviduct, but their release was inhibited; thus, the spermatozoa were unable to bind and penetrate the ovum [34, 48, 56]. It is worthy to note that roosters in pen-mated (natural mating) breeding system are known to reduce mating activity, and sexual arousal behaviour (libido) is strongly impaired during heat stress, presumably through dehydration and alteration in secretion of sex hormones. 

It has been reported that the processes of capacitation and hyperactivation in spermatozoa cells are stressful, even under normal physiological conditions [62, 63]. Reactive oxygen species, such as the superoxide anion, are produced during the important pre-requisite process of capacitation, which has been reported to occur in the female genital tract to prepare the spermatozoa for interaction with the oocyte in all species, including roosters. During this preparatory step, the levels of intracellular calcium, reactive oxygen species, and tyrosine kinase are activated, leading to an increase in cyclic adenosine monophosphate, which facilitates hyperactivation of spermatozoa. This results in increased activity of the mitochondrial respiratory chain that catalyzes motility [63–65]. Low levels of reactive oxygen species have also been shown to be essential for fertilisation, acrosome reaction, hyperactivation and motility [66]. Therefore, heat stress constitutes additional metabolic burden to an already stressful condition of the essential physiological requirements for fertilisation. This implies that heat stress may severely reduce, block, or denature binding proteins on the sperm surface that are necessary for binding to oviductal epithelium before transport to site of fertilisation [62, 67, 68].

### 4.5. Spermatozoa Structure and Peroxidative Damage

Chicken spermatozoa have unique structure and chemical composition. It has been reported that the most important feature of lipid composition of the avian semen is the presence of extremely high proportion of polyunsaturated fatty acids such as arachidonic and docosatetraenoic acids in the phospholipid fraction of spermatozoa [49, 50, 69]. There is considerable evidence that lipid composition of spermatozoa membrane is a major determinant of motility, cold sensitivity, and overall viability [70–73]. This fragile characteristic feature of the chicken spermatozoa predisposes them to peroxidative damage and associated spermatozoa dysfunction, resulting from adverse physiological conditions, including heat stress. Reactive oxygen species generated during heat stress attack polyunsaturated fatty acids in the cell membranes, which leads to a sequential milieu of chemical reactions called lipid peroxidation [74, 75]. It has been well documented that the species, that is, a compound carrying an unpaired electron, induces chain reactions with another compound to generate an unpaired electron, such that radical begets radical. These reactions are accomplished through three main steps, namely, initiation, propagation, and termination [64, 66, 76–78]. The adverse physiological reactions, culminating in lipid peroxidation in cytomembranes induced by free radicals generated during heat stress have been reported in avian species [39, 50, 51]. It is worthy to note that spermatozoa, like any other aerobic cells, are constantly exposed to the “oxygen equation,” in that oxygen is essential to sustain normal physiological life process. However, the break-down products of oxygen such as reactive oxygen species are toxic to cell functions and survival [79–81].

## 5. Environment, Season, and Reproduction Interaction

Although research on environment-reproduction interaction in domestic fowl in the Northern Guinea Savannah zone of Nigeria is scanty, the reports of the studies conducted in the zone by Obidi et al. [10, 82] support the findings of other authors [33, 50] on heat-stress infertility in roosters in the temperate zone. Bah et al. [83] documented the deleterious effects of heat stress on semen characteristics in local breeder cocks in the Sahel region of Nigeria, which depreciated the qualitative and quantitative seminal characteristics and resulted in lowered fertility. The report of Obidi et al. [10] also revealed that a significant proportion of ejaculated spermatozoa was morphologically deformed during the hot-dry season. Such sperm abnormalities include microcephalic, bent head, broken mid-piece, and cytoplasmic droplets. The sperm abnormalities have been reported to be important contributory factors to heat-stress-induced infertility in avian species [84]. 

Onuora [85] documented the effects of season, and heat stress in particular, on seminal characteristics in the guinea fowl in the warm-wet, rainforest zone of Nigeria and demonstrated that heat stress deterred optimum fertility in the zone due to a compromise in seminal characteristics. Onuora [8] further reported deleterious effects of environmental factors, including high ambient temperatures in the laying pattern of domestic hens in the same zone. Machebe and Ezekwe [86] showed seasonal variations in the ejaculate quality of Nigerian local cocks in the same zone. Seasonal variations in seminal quality and hormonal secretion have been reported in exotic avian species by Penfold et al. [84]. Rekwot et al. [87] demonstrated significant depression in quantitative and qualitative parameters of the spermiogram of Rhode Island Red breeder cocks in the Northern Guinea Savannah zone of Nigeria. The results showed convincingly that heat stress exerts deleterious effects on fertility. The report of Obidi et al. [10] also confirmed that a significant depression in spermatozoa parameters occurs during the hot-dry season, compared with that obtained during the Harmattan or rainy season. 

Obidi et al. [82] further showed the complex interplay between the environment and optimum reproductive capacity in breeder birds by inseminating breeder hens with pooled semen collected in the morning and afternoon hours to determine the effect of timing of artificial insemination on fertility and hatchability. In the study in which breeder hens were inseminated at 10:00 h and 15:00 h for four weeks, it was observed that breeder hens inseminated in the morning hours had a significantly higher fertility and hatchability than those obtained in inseminated hens during the afternoon hours. The report suggested that although endogenous factors were involved in the reproductive capacity of breeder chickens, vital environmental factors such as ambient temperature value and its diurnal rhythm [88] constitute vital exogenous parameters that play important roles in reproductive phenomena in domestic chickens. Thus, Onuora [85] reported a greater number of spermatozoa in guinea fowls when semen was collected between 18:00-19:00 h than during the mid-day. The reason for this finding may be due to the association between semen production and rhythmic changes in metabolic rate; the rate of spermatogenesis may vary with diurnal changes in body temperature as the period of highest mating activities has been found to coincide with that of the greatest semen yield [89, 90].

## 6. Heat Stress and the Incubation Process

### 6.1. Incubation Temperature and Embryonic Development

The modern incubator is a simulated artificial design that mimics the mother-hen's role (biomimetics) of providing fertile eggs with optimum environmental conditions (temperature and humidity) to stimulate embryonic development until hatching [91]. It has been demonstrated that the optimum environmental conditions are synonymous with incubation temperatures, which determine the efficiency of embryonic and posthatch development of chicks [92, 93]. Romanoff [93] and French [91] reported deleterious effects of heat stress on the incubation of the avian embryo, which agree with the reports of Hill [94] and Lourens et al. [95], who showed extensive influences of temperature on chicks' embryo development, and that environmental temperature is the most important factor in incubation efficiency.

### 6.2. Incubation Temperature, Hatchability, and Posthatch Development

Deeming and Ferguson [96] and Wilson [97], reported the effect of temperature on hatchability. Lundy [98], Wilson [97] and Lourens et al. [95, 99] confirmed the adverse effects of temperature on posthatch development of chicks. It has been shown that a constant incubation temperature of 37.8°C, established as thermal homeostasis in the chick embryo [100], gave the best embryo development and hatchability [95, 97]. Any marginal deviation from this fragile balance is detrimental to the developing embryo [95]. Thus, a constant high temperature of 38.9°C during incubation initially accelerates embryonic growth, utilisation of nutrients and energy from the yolk and albumen reserves, but later decreases embryonic development as a result of limited metabolic process by insufficient exchange of oxygen [99, 101]. 

Heat stress in the incubation process has been shown to have diverse detrimental influences on embryos. Ande and Wilson [102] showed that dead embryos occurred soon after subjecting them to heat stress, especially on days 7 and 19. This implies that embryos at these stages of development may be very sensitive to all types of stress, including heat stress, which could be related to the chorioallantoic membrane susceptibility to environmental stress.

The deleterious effect of heat stress on embryo survival was further elaborated in turkey eggs by French [103], who demonstrated that turkey eggs incubated at 38.9°C had poor embryo survival. In the report, heat-stressed embryos had increased mortality between 15–20 days and 23–25 days of incubation, which partly agrees with the report of Ande and Wilson [102] that high incubation temperature is inimical to embryo survival. In both cases, increased embryonic death is, apparently, due to increased endogenous (metabolic) heat production. The reports are in agreement with the findings of Lourens et al. [99], who documented significant embryo mortality and, hence, lower hatchability in chicken eggs, when they were subjected to a high incubation temperature of 38.9°C. Apart from embryonic mortality, the quality of chicks from heat-stressed embryos has been reported to be adversely affected. Hill [94], Lourens et al. [95, 99], and Hatch Tech [104] established that the major indices of chick quality assessment such as chick length, reported to have high correlation (*r* = 0.36) with chick quality and posthatch liveweight gain, are depressed [94]. The authors further showed that yolk-free body weight was reduced by high environmental temperature, resulting in lower percentage of first-grade chicks, thus agreeing with the findings of Decuypere and Michels [105], who showed adverse effects of heat stress on chick quality and production.

### 6.3. Chick Abnormalities due to Abnormal Incubation Temperature

Disproportionate development, circulation disruption, abnormalities, and depression in growth rate are common consequences of high ambient temperature [102]. Ande and Wilson [102] further demonstrated that hyperthermia inhibited embryonic growth rate, which resulted in increased incidence of malformation and lowered hatch weight. Heat stress during incubation depressed liver function and development [106]. High environmental temperatures were shown to induce high mortality due to ascites [107, 108]. Decuypere and Michels [105] found that overheated turkey embryos had high incidence of subcutaneous haemorrhage, chorioallantoic haemorrhage, opaque eye disc, oedematous head, excess albumen and upside-down embryos with heads positioned between the legs. 

The incidence of adverse effects of heat stress on embryonic growth has been reported [109]. Yalcin and Siegel [110] showed that over-heating fertile eggs during incubation resulted in differential tissue growth at different stages of incubation. The finding further showed asymmetries in skeletal development during the early and late stages of embryo development. Heat-stressed embryos, in the report, showed shorter face length and low lung weight, resulting in weaker chicks with high incidence of culled-out birds due to unsteady gait. The findings also support the observation of Obidi (2002, unpublished data), that high incidence of chick deformities and increased susceptibility to handling stress (such as chicks' sorting, counting, boxing and transport stress) culminate in significantly higher early life mortality of affected day-old-chicks. A greater number of culled chicks as a result of unthrifty behaviour (lower Pasgar score) (Pas Reform, [111]) in the destination farms was also reported. All the anomalies were due to heat stress suffered by embryo during the incubation process, induced by poorly controlled machine and environmental temperature due to frequent incubator electrical power failure. It has been documented by Ande and Wilson [102] that under electrical power failure, which may frequently occur in Nigeria, additional disruption of the control of ventilation and turning systems of the incubator further complicate heat stress due to lack of oxygen, excess carbon dioxide, lack of turning, and improper humidity.

Incubation temperature affects general metabolism of chicks [107]. An increase in environmental temperature may cause metabolisable energy to be diverted from growth and development to functions involved in homeothermy. High environmental temperatures reduce thyroid function and, consequently, metabolic rate, oxygen consumption, and growth rate [106, 112]. Christensen et al. [113] showed that the chick embryonic thyroid plays a major role in maturation of vital tissues during the final stages of *in ovo* life; the authors reiterated that the embryonic thyroid had a significant control of hatching times and survival rates of neonates. This paper corroborates the findings of Deeming and Ferguson [96] and Lourens et al. [95, 100], who showed that retarded embryonic and posthatch chick developments are due to consistent heat stress. Moraes et al. [112] observed lowered respiratory quotient, reflecting increased fatty acid oxidation or gluconeogenesis in response to energy deficiency in embryos during heat stress.

It is worthy to note that breed and strain variations affect responses of avian species to heat stress. Thus, French [103] and Meijerhof and Albers [107] have reported significant embryonic responses to heat stress in the embryos of broiler and layer chickens, which also differed from responses to heat stress in wild avian species.

## 7. Concluding Remarks

In conclusion, heat stress exerts poor well-being effects on the domestic chicken, and complex interplay exists between the environment and reproductive performance of the domestic chicken. Heat stress may influence the rate of secretion of hormones and their metabolic clearance rate. In addition, it may affect the sensitivity of gonads to metabolic hormones by altering the receptor numbers. The effects should be considered in future experiments, designed to elucidate the mechanism of heat stress on reproductive efficiency in the chicken, reared in the tropical and sub-tropical regions of the world. The molecular mechanisms underlying the action of heat stress on the decline of fertility, hatchability, and general welfare of domestic chickens have not been fully understood. This requires further investigation, and the elucidation of the mechanisms may facilitate the adoption of comprehensive preventive and control measures of combating heat stress in birds, reared in hot-humid zones where the thermal environment is not artificially regulated. Deleterious effects of heat stress impair embryonic physiology permanently [100, 109, 110]; these negative impacts leave a long-term residual impairment on chick quality. Measures aimed at ameliorating the adverse effects of the heat stress on embryo development are of paramount importance for profitable chick production in the tropics and subtropics.
